# Recent Developments in Surgical Treatment of Spinal Deformity in Pediatric Patients: Experience from a Single-Center Series of 42 Neurofibromatosis Type 1 Patients

**DOI:** 10.3390/cancers16234079

**Published:** 2024-12-05

**Authors:** Kiril V. Mladenov, Ralf Stücker

**Affiliations:** 1 Pediatric Orthopedic Department, Altona Children’s Hospital, Bleickenallee 38, D-22763 Hamburg, Germany; ralf.stuecker@kinderkrankenhaus.net; 2 Department of Orthopedics, University Medical Center Hamburg-Eppendorf, D-20246 Hamburg, Germany

**Keywords:** spinal deformity, neurofibromatosis type 1

## Abstract

Background: The management of spinal deformities in patients with NF-1 is challenging. The study aimed to assess the outcomes of the surgical treatment of spine deformities in children with neurofibromatosis type 1 with our treatment approach. Methods: A retrospective single-center study on pediatric patients with spinal deformities associated with NF-1 who received surgical treatment between 2006 and 2024. Results: The study group comprised 42 patients with a mean age at surgery of 9.8 years. Twenty-five patients (60%) were treated by means of growth-preserving techniques and 17 patients (40%) by means of definitive fusion. Preoperative halo-gravity traction was used in 14 (33%) cases. In the group treated with a growth-preserving technique, a 54.1% mean curve correction was observed at the latest follow-up, and growth of the thoracic spine was maintained at a physiological rate; however, 25 unplanned revision surgeries (mostly due to mechanical complications) were necessary. In the group treated by definitive fusion, a 66% curve correction was achieved at initial surgery, which remained unchanged at latest follow-up, and revision surgery was performed in three cases for augmentation of the fusion mass. There was one neurological complication (2%). Another patient developed a deep wound infection (2%). Conclusions: Good and sustainable surgical correction of spinal deformities can be achieved in pediatric patients with NF-1. Due to the bony dystrophic changes, surgical treatment is challenging and the complication rate is higher than in spinal deformities of other etiologies.

## Simple Summary

Approximately 60% of the patients with neurofibromatosis type 1 (NF-1) demonstrate involvement of the skeletal system, with the spine being the most commonly affected element. In the vast majority of the cases with an NF-1-associated spinal deformity, the natural history is unfavorable and worsening is observed in cases that proceed without treatment. Non-operative management is rarely successful and surgery is necessary in most of the cases, especially in the presence of the characteristic osseous dystrophic changes. Since the disease is rare and features a heterogeneous presentation, evidence-based treatment protocols are not available. The aim of this retrospective study is to share the experience and to report the outcomes after treatment of spinal deformities in pediatric patients with NF-1. The results show that good and sustainable correction of spinal deformities can be achieved by means of early surgical treatment even in immature patients. However, surgery remains challenging, and the complication rate is higher than in patients with spinal deformities of other etiologies.

## 1. Introduction

Neurofibromatosis type 1 (NF-1) is one of the most common genetic disorders in human beings, affecting 1/3000–4000 individuals. An estimated 1,000,000 persons are affected worldwide without gender or racial predisposition [1,2,3]. Spinal deformities in patients with NF-1 are considered the most common skeletal manifestation in NF-1 patients and have been reported in up to 60% of the affected individuals [1,4]. Typically, spinal deformities are very severe and rigid and are accompanied by characteristic NF-1 dystrophic osseous changes with involvement of the vertebrae and/or the ribs [5,6], which make treatment very challenging [7]. Non-operative approaches, such as observation and bracing, can be applied only for a very small number of patients with mild non-dystrophic “idiopathic like” curves [5,6]. In opposition to this, surgical treatment is advocated in most of the patients presenting with dystrophic spinal deformity due to the history of such cases featuring constant worsening [8,9]. Despite the development of modern surgical techniques for stable spinal instrumentation, the complication rate after surgical correction of spine deformities remains relatively high due to biological and mechanical factors. Spinal instrumentation may be very challenging due to dystrophic vertebral body and lamina, as well as absent or tiny pedicles, dura ectasia and soft bony structures. The most common complications are delayed union, pseudarthrosis, curve progression, implant failure and perioperative bleeding, which have been reported at a rate of up to 64% [10,11]. Current treatment strategies have been proposed by some authors [12,13]; however, well-established evidence-based treatment protocols, especially for the pediatric population, are still lacking. The current study aimed at evaluation of the results after treatment of spinal deformities in children with NF-1 with our treatment protocol, highlighting pitfalls and presenting different approaches depending on curve morphology and patient-specific factors.

## 2. General Knowledge on NF-1

### 2.1. Genetics, Pathogenesis and Diagnosis

Neurofibromatosis type 1 is caused by a defect in the tumor suppressor gene located on the long arm of chromosome 17, which encodes production of the ubiquitously present protein neurofibromin, its major function being the downregulation of the RAS protein [14,15,16]. NF-1 is classified as a “RAS-opathy”, which is a group of clinically related disorders that includes Costello syndrome, Noonan syndrome and gingival fibromatosis. Lack of neurofibromin results in increased RAS signaling and the development of NF-1-associated neoplasms such as peripheral nerve sheath tumors, pheochromocytoma, myeloid dysplasia and neurofibromas [17,18]. Since downregulation of the RAS follows the protein kinase pathway, protein kinase inhibitors such as Trametinib underwent clinical trials, with promising results [19,20,21]. Data on the positive effects of medical treatment on the development of spinal deformities are currently not available.

Inheritance is autosomal dominant; however, 50% of the cases are “de novo” mutations, and genetic identification of mutation can be achieved in 95% of the affected patients [22]. Since the gene is very large and affection is heterogeneous, clinical expressivity between patients is highly variable and no clear genotype–phenotype correlation exists, which makes the predictive value of the genetic testing concerning clinical presentation unreliable.

### 2.2. Clinical Presentation of NF-1

NF-1 is a multisystem disorder affecting the skin, the peripheral nerve sheaths and the cardio vascular system. Cognitive disabilities are present in 60–80% of the children [23], and skeletal manifestations, such as scoliosis, kyphosis and tibia dysplasia (congenital tibia pseudarthrosis), have been documented in up to 60% of the affected individuals. Although the disease is mostly fully present by the age of 5 years, phenotypic appearance and presentation may be highly variable between individuals and even between relatives. Typical skin manifestations are the “café-au-lait” hyperpigmented macules, the axillary and inguinal freckling, the cutaneous neurofibromas and the plexiform neurofibromas. About 10% of the patients develop malignant peripheral nerve sheath tumors (MPNSTs) with a median time of diagnosis of between 20 and 40 years; MPNSTs have a poor prognosis and may be lethal [24,25]. Ocular manifestations, such as the pathognomonic iris “Lisch” nodules (iris hamartoma), are present in children above 6 years of age and may be seen in most adult patients by slit lamp examination. Optic glioma (a pilocytic astrocytoma) is seen in 15–20% of children with NF-1, with median age of 4.9 years at presentation. It remains often asymptomatic and may cause visual abnormalities which may progress to optic nerve atrophy and blindness in certain cases [26,27,28]. The diagnosis of NF-1 is based on clinical criteria defined by the Consensus Development Conference of the National Institutes of Health from 1987 [29]. The diagnostic criteria were recently revised in order to add the importance of genetic testing as a diagnostic tool supporting the diagnosis of NF-1 and differentiating it from similar conditions with overlapping clinical presentation [30].

### 2.3. Spinal Deformity in NF-1 Patients

Spinal deformity (scoliosis, kyphosis) is the most common musculo-skeletal manifestation in NF-1 patients. The high incidence was described more than a century ago [31], and the real prevalence in NF-1 patients is unknown. However, current observations report that over 60% of the NF-1 patients may present with spinal deformities [1,4]. The exact etiology is unknown, and current hypotheses include mesodermal dysplasia, erosion and infiltration of the bone by local neurofibromas, osteomalacia and endocrine factors [4]. Curves are mostly localized in the thoracic spine, but the cervical and the lumbar spine may be involved as well [32,33]. Depending on curve appearance and radiologic bone morphology, curves can be differentiated in terms of being “non-dystrophic” and “dystrophic” deformities [4,6]. The differentiation between those two presentations is very important since deformity behavior, prognosis and management are largely different.

#### 2.3.1. Non Dystrophic Spinal Deformity

Non-dystrophic coronal curves (scoliosis) are also called “idiopathic like”; they typically present without morphological changes in the single vertebral elements (absent signs of osseous dysplasia) and behave similarly to idiopathic scoliosis. The prognosis is favorable, and the mainstays of treatment are observation for curves <20° and brace treatment for curves 20–40°, usually with a Cheneaux-type brace [6]. Particular attention should be paid to these patients, and regular clinical and radiologic curve monitoring in 6–12 month intervals is mandatory since dystrophic features may occur over time and may be followed by transformation into dystrophic deformities—a process called “modulation” [34].

#### 2.3.2. Dystrophic Spinal Deformity

**Figure 1:**
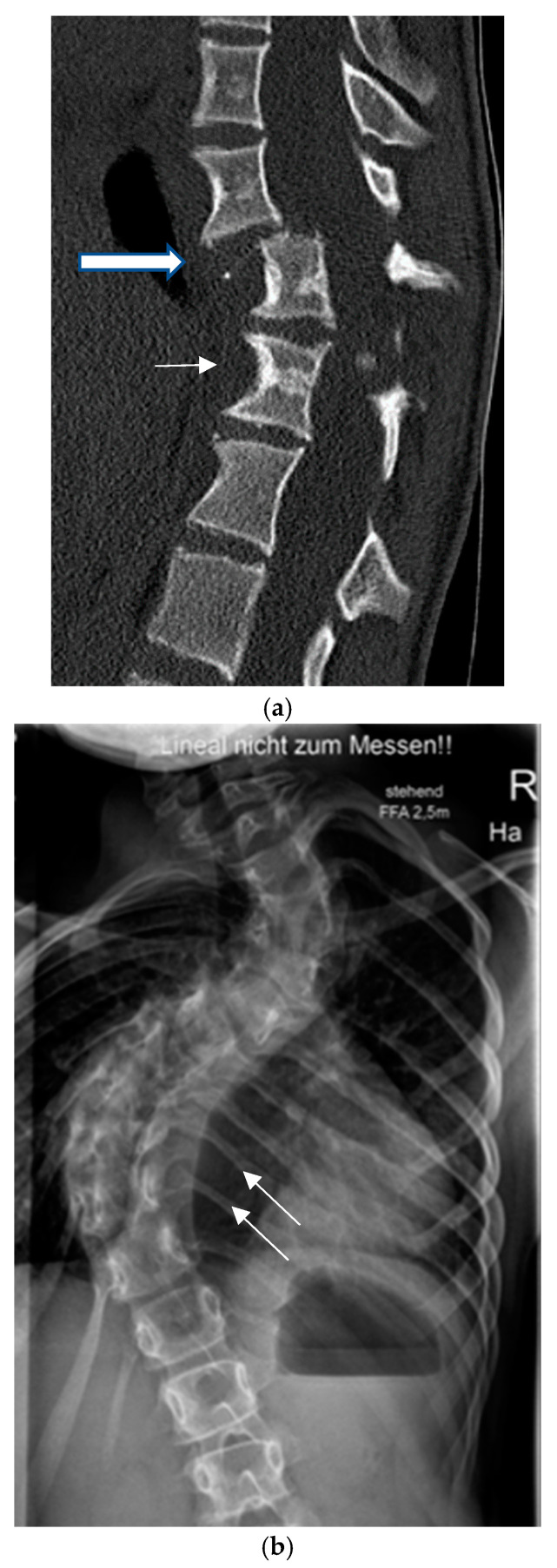

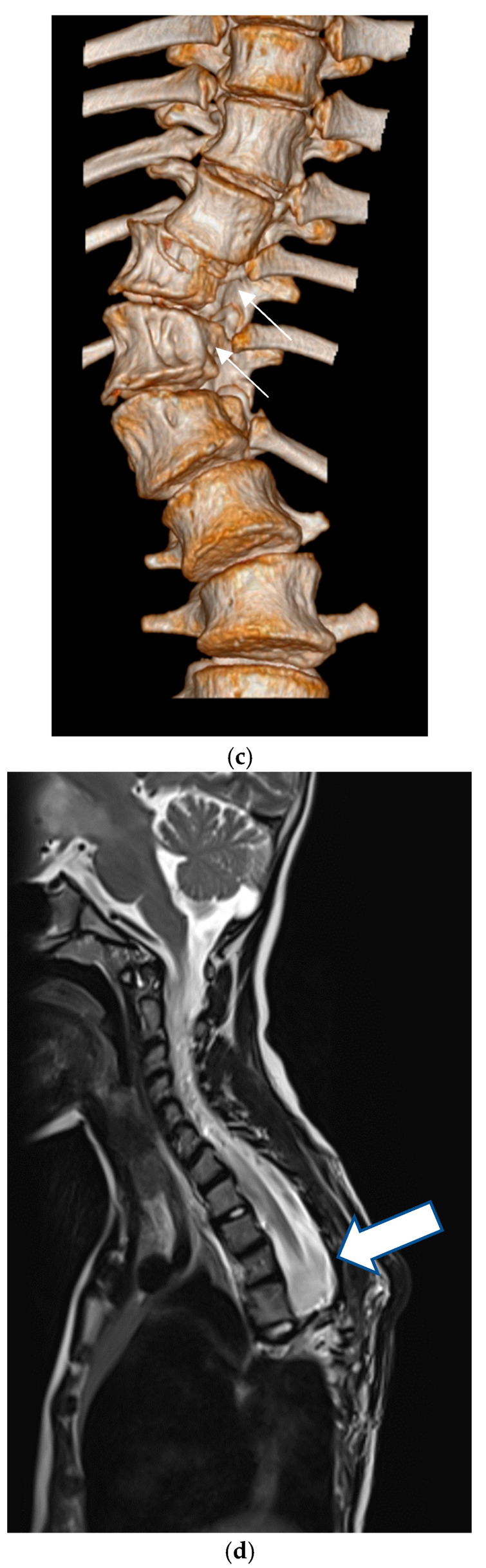
Dystrophic osseous features: (**a**) scalloping (thin arrow), rotation and dislocation with pathologic kyphosis (thick arrow), (**b**) rib penciling (white arrows), (**c**) wedging and rotatory dislocation (arrows) in a 3-D CT reconstruction, (**d**) widening of the spinal canal and dural ectasia (thick white arrow).

Dystrophic osseous changes are best seen on upright plain whole-spine radiographs, and meticulous evaluation for dystrophic features should be performed since management and prognosis of spinal deformity are highly dependent on the presence of dystrophic signs. Characteristic dystrophic features are listed in Table 1 and extensively described in Figure 1.

Dystrophic curves are typically short, extending over 3–5 spinal segments with poorly visible vertebral structures and a significant spinal rotation which occasionally leads to rotatory subluxation or complete dislocation, frequently accompanied by pathologic kyphosis (Figure 1a). It has been hypothesized that dystrophic changes are either intrinsic (due to primary bone deformation) or appear secondary to increased pressure from dural ectasia or nerve root enlargement, leading to scalloping or neuroforaminal enlargement [35]. The presence of dystrophic changes is of very important prognostic value. As a general rule, the more pronounced the dystrophic changes, the more unfavorable the prognosis. The presence of three or more dystrophic features has been related to worsening of the spinal deformity in over 85% of the cases [34].

#### 2.3.3. Imaging Modalities of Spinal Deformity in NF-1

Upright whole-spine antero-posterior and lateral plain radiographs are the gold standard for the evaluation of spinal deformity in NF-1 patients. These allow for exact measurements of the severity of the coronal and the sagittal deformity by means of the Cobb angle and the evaluation of dystrophic changes. Whole-spine MRIs are considered essential in the evaluation of spinal deformities in NF-1 patients and have become mandatory in the preoperative assessment of all patients in order to detect intraspinal and paravertebral pathologies in recent years. A combined incidence of paraspinal and intraspinal tumor pathologies has been reported in 37% of the patients with non-dystrophic and dystrophic curves [13].

Bone morphology can be best evaluated by means of a CT scan. Despite concerns of x-ray exposure, a thin-slice CT scan with coronal, sagittal and 3-D reconstructions is very valuable for the preoperative planning and for the evaluation of the fusion mass. The scan surface should be restricted to the area of interest, which mostly corresponds to the dystrophic portion of the spine in order to reduce the negative effects of radiation exposure.

## 3. Materials and Methods

### 3.1. Data Collection, Measurements

A retrospective study with prospective data collection of all patients who received surgical treatment for spinal deformities associated with NF-1 in a single institution between 2006 and 2024. Patient charts, available medical records, spinal radiographs, and other imaging studies (MRI, CT) were used for data collection.

Initial upright spine radiographs were used for the analysis of location and type of spinal deformity (scoliosis, kyphosis) as well as the presence of osseous dystrophic changes already listed in Table 2. The amount of coronal and sagittal spinal deformity were evaluated by means of standardized measurement of the Cobb angle on upright radiographs performed before surgery on the first erect postoperative radiography and at the latest follow-up. Patients were divided into groups according to the location of deformity (cervical vs. thoracic and/or lumbar), type of procedure (growth-preserving vs. definitive Fusion), and type of surgical approach (anterior, posterior or combined). In the patient cohort treated by means of a growth-preserving technique, the T1-12 height was measured on the first postoperative index image and ultimate AP radiographs in order to evaluate spinal growth. Based on the cumulative height gain during the observation period, the T1-12 growth ratio was calculated and presented as millimeter per year. Achieved correction at final follow-up was calculated as percent from the initial amount of spinal deformity. Presence of spinal fusion in the group treated by means of definitive spondylodesis was estimated on the latest available imaging study (x-ray, MRI, CT).

Information about the number of surgical procedures, the necessity for planned or unplanned revision surgery as well as perioperative and late complications was collected from patients’ records.

Demographics are reported in mean and SD values; a paired students *t*-test was used for evaluation of achieved curve correction using SPSS software, (Version 29.0.0.0) and the statistical significance was set at 0.05.

### 3.2. Treatment Protocol

Our treatment protocol is based on patient-specific and deformity-specific factors such as bone age, skeletal maturity curve location, magnitude and flexibility, as well as the presence of dystrophic osseous changes. The currently widely accepted bone age of <10 years was chosen as a treshold to define “early onset” spinal deformity as proposed by the SRS. [36]. The Sanders classification was used for the definition of bone age, with Sanders stage 7b defining end of spinal growth [37].

With respect to location, spinal deformity was divided into cervical, thoracic, thoraco-lumbar, lumbar and lumbo-sacral. According to the magnitude of coronal deformity (scoliosis), curves were divided into mild (20–40°), moderate (40–60°) and severe (>60°). With respect to osseous dystrophic changes, we divided spinal deformity into “non-dystrophic” and “dystrophic”. The treatment strategy is presented through the following case scenarios:

#### 3.2.1. Non Dystrophic Spinal Deformity

Spinal deformity in the absence of osseous dysrtophic changes in patients with NF-1 behave like idiopathic spinal deformities, and spinal curves are called “idiopathic like” [13]. Management follows the guidelines for idiopathic scoliosis treatment. Simple observation is sufficient in soliosis <20° independent of patient maturity, and the prognosis is generally good, especially after completion of skeletal growth. Immature patients with curves of 20–40° are treated with a brace until the end of growth. Curves above 50° Cobb usually need surgical treatment since worsening is observed in most of the untreated cases. The method of choice depends on skeletal maturity, with growth-preserving techniques being adequate for immature patients and definitive fusion being the recommended method after the completion of growth. Particular attention should be paid in these patients, and follow-up radiographs should be performed once a year and meticulously evaluated for dystrophic osseous signs since those may develop with time in initially non-dystrophic vertebral bodies.

#### 3.2.2. Dystrophic Spinal Deformity

The risk of deterioration of spinal deformity in the presence of dystrophic osseous changes is high and increases proportionally with the severity of bone dystrophy. Brace treatment is not efficient to stop deformity progression and is not recommended in these cases. Our approach in immature patients is to perform an anterior fusion at the apex of the deformity, which usually extends over 2–4 segments, and then perform a spine-based growth-preserving instrumentation through a posterior approach in order to correct and control the global spinal deformity. After the end of growth, instrumentation is converted to the final spinal fusion. Skeletally mature patients with scoliosis 40–60° Cobb at initial presentation underwent posterior spinal fusion (PSF) with segmental instrumentation. Patients with very severe dystrophic curves (>60° Cobb) at index presentation were treated by means of combined anterior and posterior spinal fusion.

### 3.3. Surgical Treatment Methods

#### 3.3.1. Anterior Spinal Fusion

**Figure 2:**
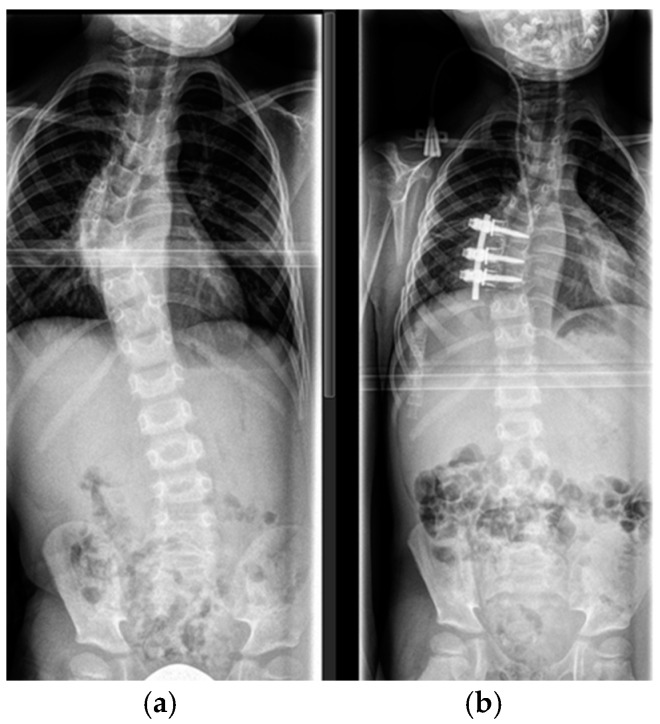
(**a**) Six-year old patient with dystrophic scoliosis of the thoracic spine. (**b**) After anterior spinal fusion Th 7–9 with instrumentation.

The rationale of anterior spinal fusion in dystrophic curves is to control the deformity at the apex of the curve through achievement of a solid fusion and preservation of the stability of the spinal column, thus avoiding the risk of neurological damage of the spinal cord. This is especially important in cases with pathologic kyphosis or rotational dislocation. Depending on the levels to be addressed, we performed the anterior part of the procedure through an open transthoracic, a retroperitoneal or a combined approach from the convex side. In the thoracic spine, anterior fusion was performed through a convex-sided thoracotomy using the intercostal space 2 levels above the apex of the curve. A partial resection of the corresponding rib was performed and the rib was preserved as a bone graft. The apical intervertebral disks (usually 2–4 segments) were meticulously removed, generously exposing the subchondral vertebral end plates. Violation of the cancellous bone of the vertebral end plates was avoided in order to avoid severe uncontrolled bleeding. A part of the rib graft was morselized and filled in the intervertebral disk spaces. An anterior screw–rod instrumentation was used, as shown in Figure 2.

**Figure 3:**
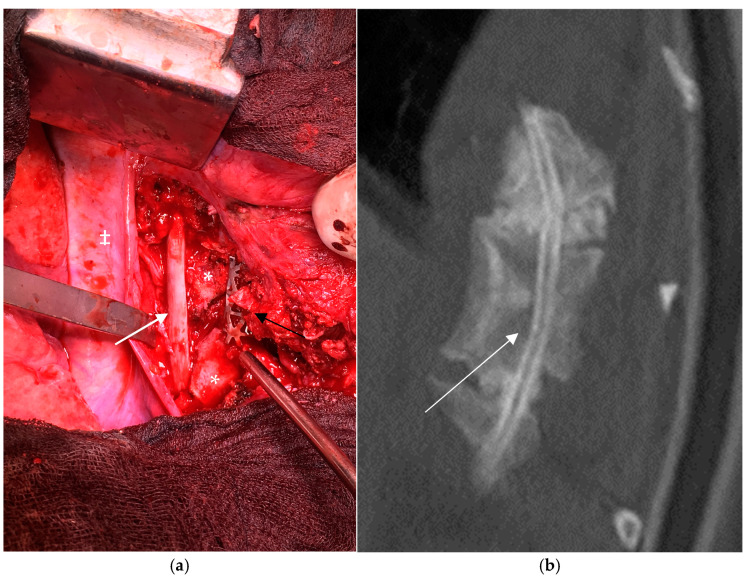
Transthoracic approach to the thoracic spine in a 15-year-old patient with dystrophic scoliosis and kyphotic deformity (**a**). Anterior strut rib graft (white arrow), anterior vertebral body support with an intercorporal mesh cage (black arrow), vertebral bodies (*), aorta (‡); (**b**) The rib graft (white arrow) in place with a bony fusion confirmed by a thin-slice CT scan.

In patients with severe dystrophic changes and insufficient bony substance of the vertebral bodies, the remaining portion of the rib was anchored as a strut bone graft in a gap created with a rongeur in the antero-lateral portion on the convex side of the apical and the juxta apical vertebral bodies, as shown in Figure 3.

In the lumbar spine, the fusion was achieved through a retroperitoneal approach, and titanium mesh cages filled with autologous cancellous bone graft from the iliac crest were used for anterior support in order to maintain physiologic lordosis. If the apex was located in the thoraco-lumbar junction, a combined transthoracic–retroperitoneal approach with diaphragmatic release was used.

#### 3.3.2. Posterior Growth-Preserving Instrumentation

Preservation of the thoracic spine growth in immature patients has been shown to play an essential role in the physiologic development of the lung tissue and is of vital importance in terms of preserving normal lung function and avoiding the development of iatrogenic thoracic insufficiency syndrome [38]. We used the following spinal growth-preserving techniques, all of which are based on active distraction:

##### VEPTR

**Figure 4:**
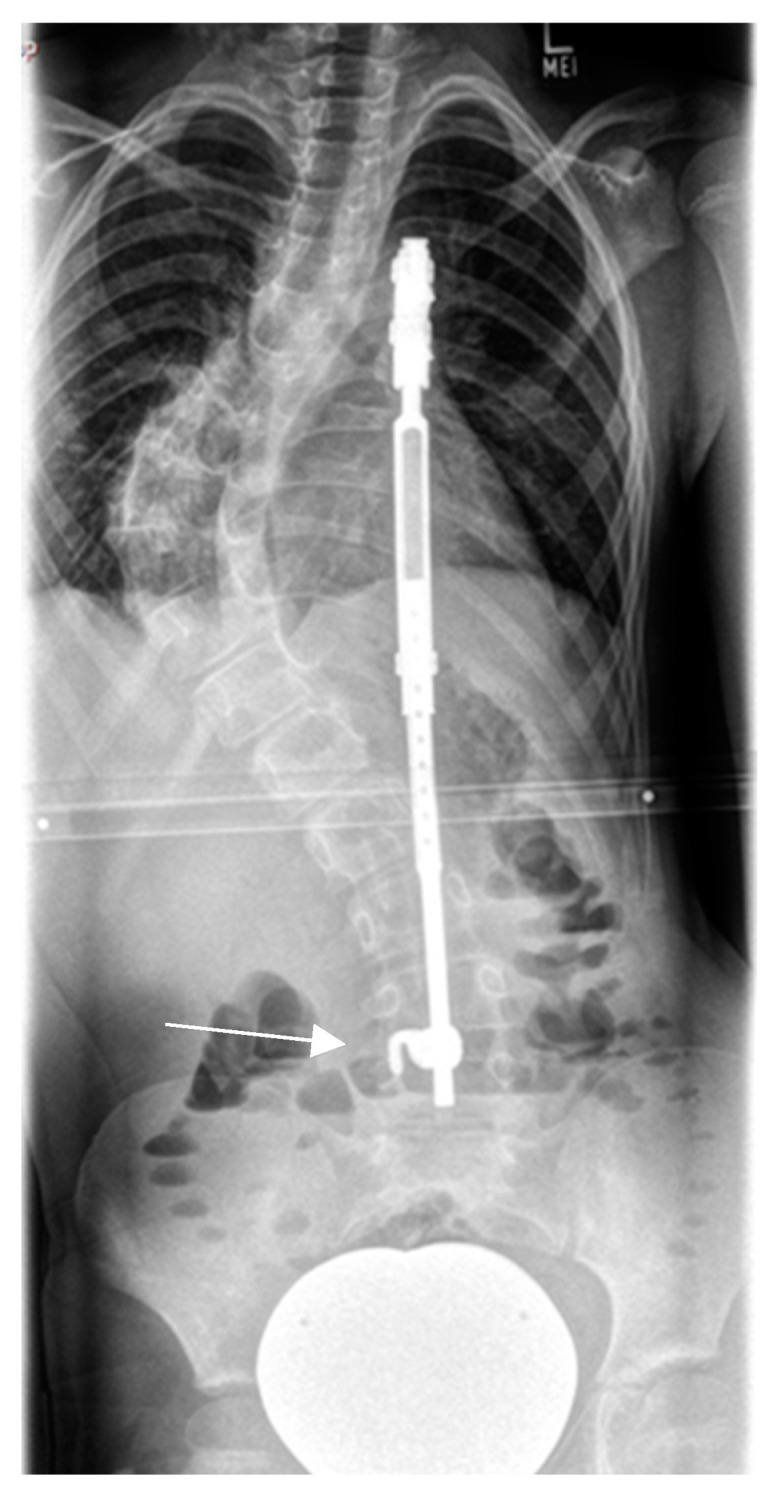
Rib-to-ilium VEPTR construct in a 9-year-old patient with a dystrophic curve. Distal migration of the laminar caudal anchor (white arrow) with loss of correction.

The vertical expandable prosthetic titanium rib (VEPTR^®^, De Puy Synthes, Raynham, MA, USA) was initially designed for the treatment of congenital scoliosis associated with rib anomalies and chest-wall deformities. Later, the device was approved for clinical use in immature patients with scoliosis of a non-idiopathic origin. The technique consists of proximal and distal anchors connected with a telescoping device which can be lengthened surgically, usually every 6 months, by a minimally invasive approach. The proximal anchors are attached to the ribs, and the distal may be attached to the ribs, directly to the spine by means of a laminar hook or to the pelvis by means of an ilium “S” shaped hook. We used this technique upon performing index surgery in an early series of immature patients; however, due to the high number of mechanical complications due mostly to aseptic loosening of the anchors which migrated through dystrophic bone its use was abandoned Figure 4.

##### Traditional Growing Rods (TGRs)

**Figure 5:**
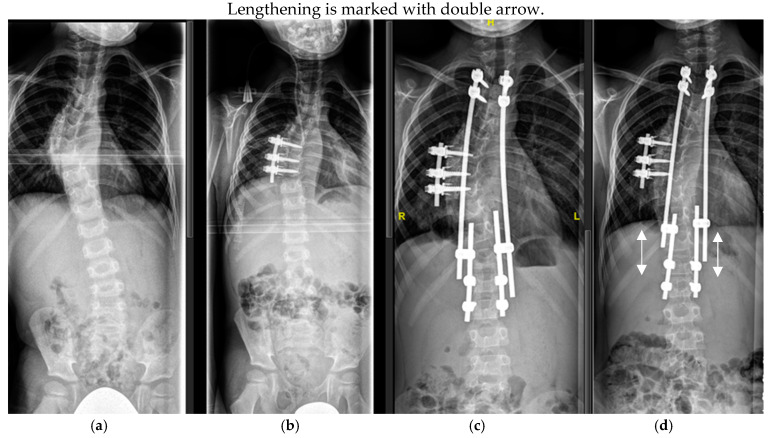
Six-year-old patient with dystrophic scoliosis (**a**). After a short-segment anterior fusion T7-9 (**b**). After TGR application T3-L2 (**c**), and after 3 years of follow-up and 5 TGR lengthenings (**d**).

The surgical technique consists of cranial and caudal anchors (laminar hooks or pedicle screws) attached directly to the spine which are placed through a less-invasive posterior surgical approach. Each anchor is connected with a rod. The cranial and the caudal rods connected to the anchors are coupled to each other with a longitudinal or a parallel connector in such a manner that instrumentation extends from the upper to the lower end vertebra of the curve. The rods are left overlapping each other for 3–5 cm, allowing repetitive lengthening of the construct between the cranial and the caudal anchor (Figure 5). In that way, control of the curve may be achieved, and further growth of the spine is possible by repetitive lengthening of the rods by means of a small surgical procedure, thus avoiding growth disturbance, which is one of the main drawbacks of definitive fusion.

##### Magnetically Controlled Growing Rods (MCGRs)

**Figure 6:**
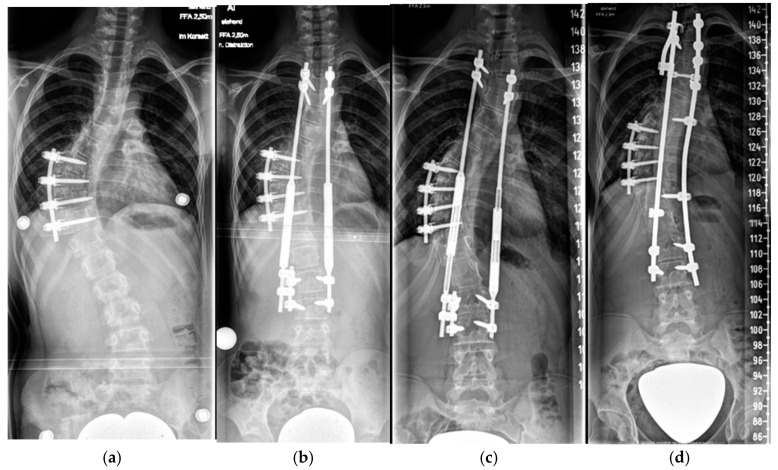
Nine-year-old patient with a dystrophic thoracic curve after short-segment anterior spinal fusion Th8-11 (**a**). After MCGR application Th3-L3 (**b**). After 5 years of follow-up and a 4.5 cm lengthening (**c**). After definitive fusion at maturity (**d**).

To avoid the necessity of multiple surgeries for rod lengthening, growing rods which can be lengthened without surgical intervention were introduced (Figure 6). The principles of anchor placement are similar to those of TGRs, but the magnetically controlled growing rod incorporates a magnet unit (“motor”) which is coupled with a threaded rod. The “motor” is activated by means of transcutaneous magnetic waves produced by an external device (called a controller) which is placed directly on the skin. In this way, non invasive lengthening of the rod can be achieved without skin incisions and without the need for anesthesia. Our lengthening protocol comprises rod lengthenings of 5 mm every 4 to 6 months, which are performed in the outpatient department. Despite the benefits of MCGRs in terms of avoiding multiple surgeries for lengthening of the device, their application is limited and practically impossible in cases which need MRI follow-ups since the magnetic unit produces a strong magnetic artifact extending 20 cm in diameter around the magnet unit, making MRI assessment of the structures located in this field impossible. Another limitation are patients with severe kyphotic deformities, since the lengthening unit of the MCGR rod should remain straight and cannot be contoured in order to match spinal deformities.

#### 3.3.3. Posterior Spinal Fusion

In this classical technique, the posterior vertebral elements are subperiosteally exposed through a posterior surgical approach. Segmental instrumentation of the vertebral column is performed using transpedicular screws or sublaminar fixation by means of hooks or bands. Spinal deformity is corrected by means of forces applied to the fixation points, which are then connected to longitudinal rods and locked in the corrected position. The facet joints are removed and the posterior vertebral elements are meticulously exposed and decorticated in order to assure good bone graft–recipient contact. Subperiosteal exposure is performed very cautiously using electrocautery in order to control bleeding and to avoid inadvertent penetration and violation of the spinal canal, especially if bone erosion is present and significant dural ectasia was seen in a preoperative MRI. This is followed by a generous bone graft application by abundant autologous iliac crest cancellous bone in order to achieve fusion.

**Figure 7:**
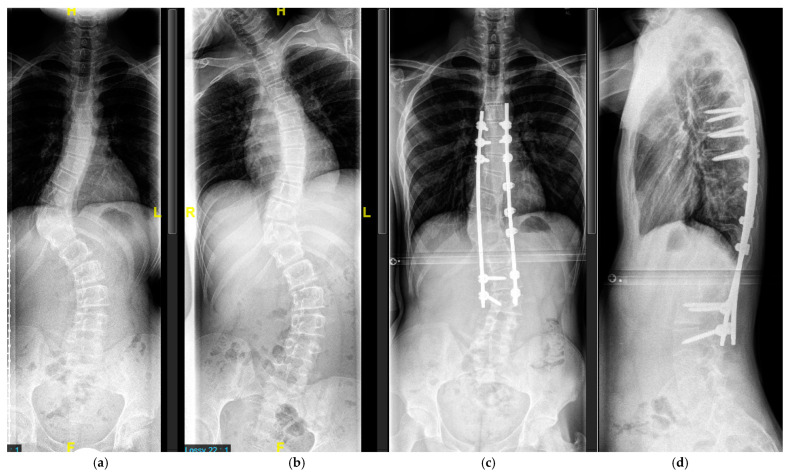
Sixteen-year-old patient with severe scoliosis of 65° Cobb (**a**). Good curve flexibility on the lateral bending film (**b**). AP (**c**) and lateral (**d**) radiographs after posterior-only spinal fusion T7-L3. Fixation in the upper thoracic and the lumbar spine with transpedicular screws, fixation in the apical dystrophic area with sublaminar wires.

Levels of posterior fusion extended from the cranial to the caudal end vertebra. Vertebra-based dual rod segmental instrumentation was used with transpedicular screws. In the presence of bone erosion and pedicle dystrophy, hooks or sublaminar bands are used, and care was taken to prevent violation of the dura (Figure 7).

#### 3.3.4. Combined Anterior and Posterior Spinal Fusion

**Figure 8:**
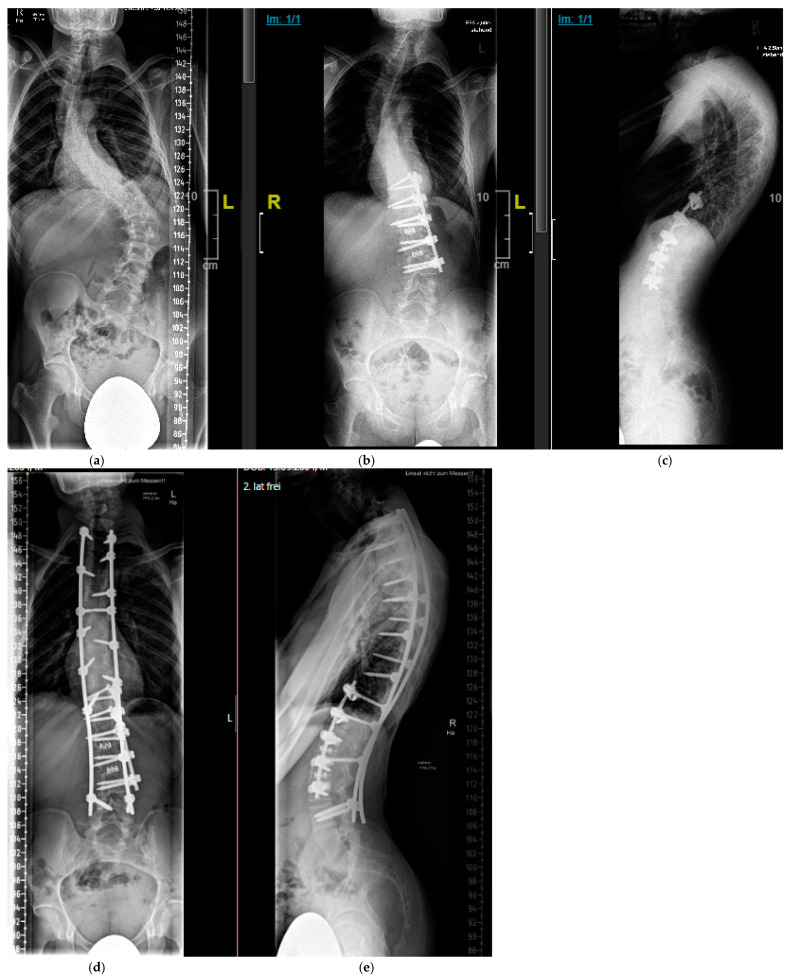
Sixteen-year-old patient with severe, rigid dystrophic scoliosis (**a**). After anterior instrumented spinal fusion T11-L3 with intervertebral mesh cages for restoration of physiologic lumbar lordosis (**b**,**c**). After combined anterior and posterior Fusion T2-L4 (**d**,**e**).

This is a combination of anterior and posterior fusion techniques. If a combined anterior–posterior spinal fusion was indicated, critical assessment of the stability of the vertebral column was performed. If the spine was stable, an anterior approach was performed as an initial procedure in order to achieve better correction through removal of the disks. In the presence of a proven or questionable instability, a posterior approach was performed first in order to achieve stability and prevent neurological injury (Figure 8).

#### 3.3.5. Halo-Gravity Traction

**Figure 9:**
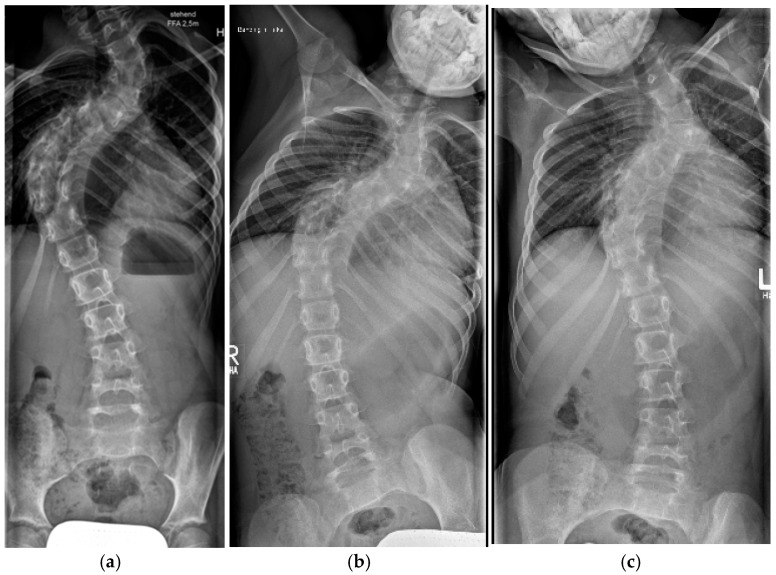

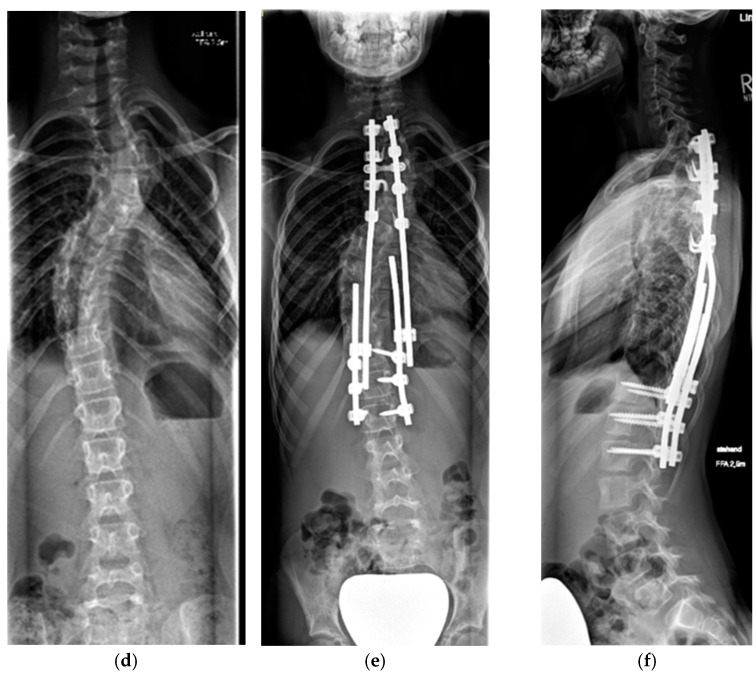
Nine-year-old patient with severe dystrophic scoliosis (**a**). Lateral bending radiographs showing curve rigidity (**b**,**c**). After 4 weeks of halo-gravity traction with excellent curve correction (**d**). After TGR application (**e**,**f**).

The method is used for temporary preoperative correction in severe, rigid, dystrophic curves independent of the age of the patient. The rationale of HGT is a slow deformity correction by means of continuous longitudinal distraction, allowing for adjustment of neurological structures and avoiding perfusion compromise of the spinal cord. Furthermore, a significant amount of the spinal deformity is reduced before surgery; in that way, no strong corrective forces need to be applied. At our institution, the patient is treated with a short general anesthesia and a Halo ring is applied to the head and fixed to the skull with 4–8 pins. Each pin penetrates just the outer skull lamina and is tightened with a torque of 3–8 Pound/Inch^2^ depending on the age of the patient. The patient is admitted to the hospital and a continuous longitudinal traction (24/24 h) is applied, beginning with 10% of the body weight and increasing up to 60% of the body weight of the patient. The patient is provided with an individually fitted wheelchair and posterior walker; additionally, they are placed in bed traction for a duration of 4 weeks before spine surgery. Vital-sign monitoring and neurologic examination (cranial nerves included) are performed every 8 h. Oxygen saturation is continuously recorded during sleep. Curve correction is monitored clinically on a daily basis and by means of serial radiographs every 7 days during the traction period. (Figure 9).

We set the following indications for preoperative HGT *:

Severe coronal curve (scoliosis) of >90 °Cobb angle

Rigid curve which does not improve <50° Cobb angle on bending film, especially in association with dystrophic osseous changes.

Severe kyphotic deformity (>45° Cobb in the cervical spine or >90° Cobb in the thoracic spine)

* Presence of at least one criterium.

#### 3.3.6. Bone Grafting

At our institution, a combination of autologous bone graft obtained from the resected facet joints and cancellous bone obtained from the posterior iliac crest combined with heterologous stripes of beta tricalcium phosphate is routinely used for cases receiving posterior fusion.

A morselized autologous rib graft is used in instrumented anterior fusion and a rib strut graft is used in non instrumented cases, as previously described.

A human recombinant bone morphogenic protein (Rh-BMP-2) is used “off-label” in cases with severe osseous dystrophy with menacing vertebral column instability and risk of spinal cord compromise in order to achieve rapid fusion and stability. Critical assessment of local morphology is performed on MRI images before application in order to avoid proximity of application to areas affected by neurofibromatosis tumor tissue. Informed consent is obtained from the official patient guardian(s) before rh-BMP-2 application.

## 4. Results

Between 2006 and 2024, a total of 42 NF-1 pediatric and adolescent patients received surgical treatment for spinal deformities at our institution. The mean age at initial surgery for the complete cohort was 9.87 years (range 4.3–17.5) and the mean follow-up was 5.28 years (range 2 months–15.1 years). At index surgery, 17 patients (40%) had definitive fusion (mean age 13.4 years; mean follow-up 3.1 years) and 25 (60%) were treated with growth-preserving instrumentation. A total of 14 patients (33%) received preoperative halo-gravity traction (HGT) based on our indication criteria. Preoperative HGT allowed for achieving very good curve correction even in the presence of severe, rigid deformity, averaging at 61%, which was similar to the correction in the non-HGT group (59,6%). Recombinant human bone morphogenic protein type 2 (rhBMP-2) was used “off label” in seven patients (at the time of the index procedure n = 3 and at time of revision or last procedure n = 4). No adverse effects related to rhBMP-2 application were encountered during the observation period. Unplanned revision surgery was needed in 18 patients (42%); in six of these patients, more than one revision surgery was indicated. In all cases, the curve was corrected and progression of deformity could be stopped. Results according to type of initial surgery are summarized in Table 2. Data are reported as a mean and (±1 SD).

### 4.1. Cervical Spine

The cervical spine was affected in three patients (7%), all of them presented with severe dystrophic kyphosis and were treated with preoperative halo-gravity traction followed by combined anterior and posterior spinal fusion. Initial cervical kyphosis measured preoperatively 97° (range 70–125°) and was corrected to 25° (range 10–52°) at latest follow-up, corresponding to a mean correction of 77% (range 59–87%).

### 4.2. Thoracic and Lumbar Spine

The patients treated for deformity below the cervical spine were divided depending on the age at which the index surgery was performed.

#### 4.2.1. “Early Onset Spinal Deformity”

Growth-preserving instrumentation was used in 25 cases representing 60% of the whole study group. The mean age at index surgery was 7.7 years (range 4.3–11.1), and the mean follow-up was 6.7 years (range 0.2–15.1). The initial growth-preserving surgical method was VEPTR in 10; MCGRs in 9 and TGRs in 6 patients. In a total of 10 cases from this group, growth-preserving instrumentation was combined with short apical anterior fusion. The Cobb angle of the major coronal curve (scoliosis) was measured immediately before surgery 77° (range 50–88°) and was corrected to 33.1° on latest follow-up, representing a 54.1% deformity correction. Immediate postoperative height of the thoracic spine (T1-12) averaged 19.6 cm (range 13.6–23.4 cm) and increased during the treatment period to 22.8 cm (range 16.8–28.1 cm) at latest follow-up, corresponding to a T1-12 growth rate of 0.73cm/year. Lengthening procedures were routinely performed at 6–9 month intervals, averaging 1.7 lengthening per patient and per year follow-up. A total of 25 unplanned revision surgeries were performed, averaging one revision surgery per patient. The most frequent reasons for revision surgery were anchor dislocation or implant failure (n = 16), as well as exhaustion of the lengthening reserve of the implant (n = 8). A deep late infection was encountered in one patient. Neurologic symptoms in association with spinal deformity in one patient who developed incomplete, flaccid lower paraparesis 10 years after index intervention for deformity correction. In this patient, kyphotic deformity worsened during the observation period due to progressive dystrophic osseous changes representing the characteristic NF-1-related “modulation process”. The patient was treated by removal of the growth-preserving instrumentation and the intermittent application of HGT for slow correction of kyphosis over 4 weeks, followed by anterior and posterior spinal fusion and anterior strut grafting in the same surgical setting. Flaccid lower paraparesis improved partially during the 6 months following the revision surgery, and the patient was able to ambulate short distances with a posterior walker at the most recent follow-up 5 years after the revision procedure. During the observation period, seven of the patients from this group reached skeletal maturity and growth-preserving instrumentation was converted to definitive spinal fusion.

#### 4.2.2. Adolescent Spinal Deformity

We performed definitive spinal fusion at index surgery in 17 patients. In nine of them, posterior fusion was performed as a “stand alone” procedure. Seven patients underwent combined anterior and posterior fusion either during the same anesthesia session or as staged procedures. The preoperative Cobb angle of the major coronal curve measured 66° and was corrected to 21° on latest follow-up, corresponding to a 66% average curve correction. No early neurologic or inflammatory complications were observed. Revision surgeries with simultaneous augmentation of fusion mass were necessary in three patients (loss of correction due to “modulation process”—1 patient; implant failure due to pseudarthrosis—2 patients).

## 5. Discussion

Involvement of the skeletal system has been reported in up to 60% of the patients with NF-1 [1,4], with the spine and the tibia being mostly involved. The management of the spinal deformities in those patients requires particular attention since most of the cases need surgical treatment, which is very challenging due to the severity of the curves, the presence of dystrophic changes and the high risk of complications, such as pseudarthrosis, bleeding and infection [12]. It is obvious that, due to the very heterogenic presentation of NF-1-associated spinal deformities, it is very difficult to create categorical divisions of the patients in clearly defined subgroups based on the morphological aspect of the deformity or on other criteria. We focused on the treatment approach depending on the skeletal maturity and the expected spinal growth of the patients studying the outcomes after growth-preserving surgery and definitive fusion methods. Since the treatment rationale of different “growth preserving” techniques is identical, our purpose was not to compare those techniques nor to compare the results in immature patients with older patients who received final fusion. Instead, we aimed at evaluation of the outcomes with our treatment protocol, focusing on spinal deformity correction, spinal growth preservation and complication rate. Since presentation and management of spinal deformities in NF-1 patients differ in relation to the affected segment of the spine, those entities will be discussed separately.

### 5.1. Cervical Spine

The cervical spine is affected in up to 30% of the NF-1 patients presenting with spinal deformities [39]. Characteristic diagnosis can easily be overlooked since patients are asymptomatic and more attention is paid to the more pronounced thoracic or lumbar deformities. The most common subjective complaint is usually neck pain. On clinical observation, a tumor mass or kyphosis may be present. The patient evaluation includes a questioning for neck symptoms and a thorough examination of the C-spine, including proof of range of motion and a complete neurologic exam. In the presence of clinical abnormalities, AP and lateral radiographs should be obtained, especially before endotracheal anesthesia or a halo-gravity traction in order to exclude instability. Evaluation of standard flexion/extension radiographs is difficult due to the complexity of the deformity, and the presence of dystrophic changes and advanced imaging MRIs or a thin-slice CT scan is recommended in most of the cases. C-spine deformities are mostly dystrophic and need surgical treatment since there is underlying or impending instability with a high risk of neurologic compromise due to spinal cord compression. The purpose of treatment is to correct deformity and to obtain solid fusion in order to preserve neurologic function. Anterior-only, posterior-only or combined fusion are discussed in the literature [40]. In our institution, a combined anterior and posterior fusion with instrumentation is the method of choice. All patients in our cohort presented with severe dystrophic kyphosis and were treated with preoperative halo-gravity traction followed by combined anterior and posterior fusion. A structural anterior support is necessary in order to restore the height of the anterior column in cases with significant dystrophic alterations of the vertebral bodies. We used a mesh titanium cage filled with autologous cancellous bone. In all our cases, a posterior fusion using autologous ilium bone graft with segmental fixation and double rod instrumentation was additionally performed. A halo vest was applied to protect instrumentation until solid union was confirmed by means of a CT scan performed 3 months after surgery. In two patients, solid fusion was achieved after the index procedure, and one of the patients showed delayed union and needed an augmentation procedure that included an autologous cancellous bone graft and “off-label” rhBMP-2 application.

### 5.2. Thoracic and Lumbar Spine

Deformities located in the thoracic and the lumbar spine are treated identically.

The natural history of spinal deformities in the presence of dystrophic osseous changes is unfavorable, with inevitable deterioration occurring in untreated cases [9,10]. Brace treatment has been shown to be ineffective in those patients, and surgical treatment is generally recommended in order to correct deformity and achieve a well-balanced, stable spine and preserve neurological function [6,41].

“Curve-specific” and “patient-specific” factors play an important role in decision-making and establishment in regard to surgical strategy.

The curve-specific factors include:

- Severity and rigidity of the deformity;
- Extent of dystrophic bone changes;
- Presence of paraspinal or intraspinal abnormalities (neurofibroma, dural ectasia, meningocele, etc.).

The patient-specific factors include:

- Expected residual growth of the spine (in particular the thoracic portion);
- Patient’s general condition and nutritional status;
- Co-morbidities such as hypertension, pulmonary insufficiency etc.;
- Accompanying malignant transformation of neurofibromas.

#### 5.2.1. Growth-Preserving Techniques

The historically well-established gold standard of treatment for dystrophic spinal deformities in NF-1 patients in order to achieve corrections and to stop deformity progression is instrumented spinal fusion. However, the negative effects of early spinal fusion in very immature patients in terms of producing growth inhibition of the thoracic spine, resulting in an iatrogenic extrinsic respiratory restrictive disease known as “thoracic insufficiency syndrome” (TIS), have been well recognized [42]. The severity of growth inhibition is directly related to the number of the fused thoracic spine segments [38]. Accordingly, as few segments as possible should be fused in an immature patient in order to preserve thoracic and lung growth. Despite this observation, consensus about the best type of procedure in early onset scoliosis NF-1 patients is still lacking. Tauchi et al. [43] reported their results after circumferential anterior and posterior spinal fusion in 11 immature patients with NF-1. Patient age at surgery averaged at 8.4 years and 67% initial correction was achieved and remained unchanged at the latest follow-up at 14 years. Solid fusion was documented in all cases. Despite observed acceptable values for lung function averaging 75.0% of the predicted amount, the observed spinal growth inhibition (T1-S1) in this study was significant and averaged only 0.39 cm/year, representing 32% of the expected amount.

Jain et al. recommended the traditional growing rods technique as a stand-alone method for the treatment of early onset spine deformity in NF-1 patients and reported an average curve correction of 51% of the initial curve [43]. In the same study, successful preservation of spinal growth (T1-S1) was observed and was measured at 1.12 cm/year. In our study, a 54.1% correction of the major curve was observed at an average follow-up of 6.7 years. We focused our evaluation on preservation of thoracic spine growth, since this is much more important for pulmonary function, and observed an average T1-12 growth of 0.73 cm/year, which does not significantly deviate from normal thoracic growth, which is reported as 0.84 cm/year (range 0.7–1.2 cm/year). Even in the patient cohort (10 out of 26 patients in our series) who received short anterior fusion in combination with growth-preserving posterior instrumentation, thoracic spinal growth was well preserved and was comparable with the subgroup without short apical fusion. Despite good deformity control, the literature-reported rate of implant-related complications with growth-preserving techniques remains very high at 57% [43]. The most common complication is proximal anchor failure, occurring in 35% of the cases [43]. Our observations are in accordance with previous reports. Of the 25 patients in our cohort treated with growth-preserving techniques, mechanical complications due to implant failure were observed in 16 patients, representing 64% of the cases. In eight patients, revision surgery was needed due to the exhausted lengthening possibility of the implant, which is an expected event and does not represent a complication in treatment. Only one patient in our series (4%) needed revision surgery due to deep infection.

It is evident that growth-preserving surgical techniques are associated with lower correction rates and higher incidence of implant-related complications for scoliosis associated with NF-1 patients as compared to definitive fusion [44]. However, thorax and lung growth preservation plays a key role in the treatment of early onset scoliosis.

Our favorite treatment approach to an immature NF-1 patient with a dystrophic curve is to perform a posterior growth-preserving instrumentation (preferably vertebra based traditional growing rods) extending from the upper to the lower end vertebrae of the major spinal curve combined with short anterior instrumented (preferred) or uninstrumented fusion extending only to the dystrophic segments. Due to the dystrophic osseous changes, a normal growth in the affected segments is not to be expected and a fusion of 3–5 levels would not lead to significant growth disturbances. Our results confirm the advantages of this approach, comprising growth preservation, achievement of stability in the dystrophic portion of the spine through solid fusion and restoration of spinal balance by straightening the spine with a concurrent restoration of thorax symmetry, which is important for the chest wall breathing excursions.

#### 5.2.2. Definitive Spinal Fusion

The treatment strategy in NF-1-associated spinal deformity after skeletal maturity aims at deformity correction and the achievement of solid spinal fusion through anterior, posterior or combined approaches.

Moderate curves (45–60° Cobb) or severe curves (>60°) can be treated by a single posterior fusion provided that the deformity is flexible and straightens to <25° upon side bending.

**Figure 10:**
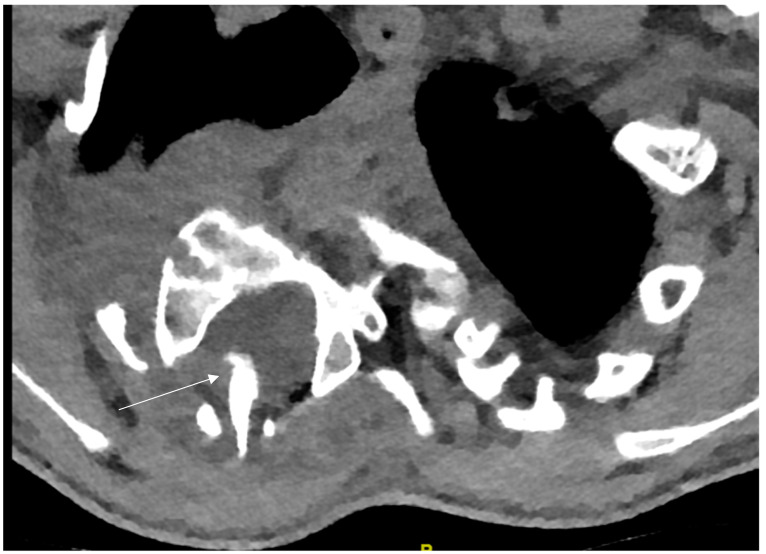
Concave rib-head protrusion into the spinal canal (arrow).

Severe, rigid, dystrophic curves (>60° Cobb) are best treated by a combined anterior and posterior fusion [8,12,45,46,47,48] which can be performed in the same or in two separate surgical settings. Due to the poor bone quality, the dystrophic osseous changes and the severity of the deformity, surgery is very challenging and meticulous preoperative evaluation of the osseous vertebral morphology, the paravertebral structures as well as the spinal canal are mandatory and of utmost importance. Besides upright native whole-spine radiographs and bending films, a whole-spine MRI should be performed in order to define critical space-occupying lesions, spinal cord compression, dural ectasia or vertebral erosion. Additionally, a thin-slice CT scan with sagittal, coronal and 3-D reconstructions is needed in order to assess the vertebral morphology and rotational deformity, as well as for the choice of instrumentation (transpedicular screws vs. sublaminar fixation). Particular attention is paid to the dystrophic apical region where osseous changes are the most pronounced. Meticulous analysis of the morphology, especially in severe cases, is mandatory. The presence of rib-head protrusion into the spinal canal through a neuroforamen, which is a typical finding on the convex side of the apical vertebra, should be ruled out since it may migrate more medially into the spinal canal during curve correction and exposes the spinal cord to high risk of compression and neurological damage (Figure 10).

Koptan et al. [12] reported on their experience with spinal fusion and dystrophic NF-1-associated spinal deformities. The authors achieved 61% deformity correction with two-staged anterior and posterior fusion using a segmental spinal instrumentation augmented with sublaminar wires. Our approach comprised a preoperative evaluation of curve characteristics in order to define the needs of the combined anterior and posterior approach. Moderate (45–60° Cobb angle) and severe (>60° Cobb angle) curves which were flexible in preoperative bending films were treated by posterior-only fusion. In contrast to this, patients with severe, rigid (unflexible) dystrophic curves received anterior and posterior fusion. In our series, definitive fusion was performed as the initial surgery in 17 patients. In nine of them, a posterior-only fusion was performed as a “stand alone” procedure. Achieved correction averaged a 66° Cobb angle, corresponding to a 66% deformity correction upon follow-up at 3.4 years. A pseudarthrosis rate after an attempted spinal fusion of up to 31% has been reported in NF-1 patients [5]. We did not observe pseudarthrosis development in our series; however, deformity progression after performed fusion associated with osseous modulation changes occurred in three cases (17%). These cases needed revision surgery for augmentation of the fusion mass.

Based on observations of delayed bone healing and pseudarthrosis after attempted fusion procedures in NF-1 patients, some authors suggested the use of biologic osteoinductive substances such as rhBMP-2 in order to promote bone union [49]. However, rhBMP-2 application is controversially discussed, and its routine use, especially in immature patients, is not recommended due to the unknown systemic effects in pediatric patients and the hypothetic risk of malignant transformation. In our series, rhBMP-2 was used on an “off-label” basis during the index procedure in the presence of severe dystrophic osseous changes and bone erosions with an insufficient contact bone surface and anticipated delayed bone union 6 months after surgery. We empirically defined “bone contact surface insufficiency” as anterior intervertebral contact of less than 1 cm^2^ and/or a bone gap between the posterior elements of more than one segment.

In the presence of severe, rigid spinal deformity, a preoperative halo-gravity traction should be considered in order to achieve sufficient correction. Our observations showed that a similar deformity correction can be achieved by means of preoperative HGT even in the presence of a severe, rigid deformity. Curve correction in the HGT group averaged 61% and was comparable to the non-HGT group (59.6%). We used rhBMP-2 in eight patients. Complications related to BMP application were not encountered in any of the patients. Despite solid fusion, which was observed in all these cases, no representative statistical data in support of the rhBMP-2 effect in promoting bone union can be presented due to the small number of patients. It should be emphasized that BMP application in the pediatric population is “off-label”. Its use should be discussed individually and the decision remains at the discretion of the surgeon. BMP application should be avoided in the proximity of existing tumor masses due to the potential risk of malignant transformation.

Since NF-1 patients present with many comorbidities, such as hypertension, intraabdominal and intrathoracic tumors, optic glioma, nutritional issues, Vitamin D deficiency [50], cognitive disabilities, etc., a multidisciplinary approach was recommended [51]. We can strongly support this recommendation and perform a preoperative multisystemic interdisciplinary evaluation of all NF-1 patients on a routine basis, which, besides orthopedic surgical assessment, also includes cardio-pulmonary, anesthesiologic, neurologic and psychological assessments in order to minimize peri- and postoperative complication risks.

The study has several limitations. Data collection was prospective, but this was not a randomized controlled study. Not all of the patients reached skeletal maturity at the latest follow-up, and some of the patients with growth-preserving instrumentation are still under treatment. However, heterogeneity of presentation and variability of surgical approaches make the feasibility of a randomized controlled study (with a random selection of either treatment or simple observation or comparison of different surgical approaches) not feasible in the current state since such a study design would face severe ethical issues, making randomization impossible. Collection of observational data on rare conditions is first needed. A strong side of the study is the uniform treatment protocol and the single-center study design.

## 6. Conclusions

Surgical correction is the mainstay of treatment for dystrophic NF-1-associated spinal deformities. This differs significantly from idiopathic scoliosis, where less than 10% of the patients need surgery.

The treatment of choice for immature patients is posterior growth-preserving spinal instrumentation from the upper to the lower end vertebra combined with short apical fusion of the dystrophic segments.

In immature NF-1 patients, spinal growth can be preserved by means of “growth preserving surgical methods” at a near physiological rate; however, the rate of revision surgery due to mechanical complications is high, with 64% of the patients needing unplanned surgery when treated with the “growth preserving” technique.

Preoperative halo-gravity traction should be considered in cases of severe rigid NF-1-associated spinal deformity. A satisfactory curve correction averaging 60% of the initial curve can be achieved, which is comparable to patients with milder and flexible deformities.

Definitive spinal fusion is recommended for patients older than 10 years. In the presence of dystrophic bone changes, the method of choice is combined anterior and posterior spinal fusion with segmental instrumentation.

As opposed to idiopathic spinal deformity, which is usually treated by instrumented posterior-only fusion, more than 50% of the patients with NF-1-associated spinal deformities need combined anterior and posterior spinal surgery in order to achieve solid fusion and avoid pseudarthrosis.

Treatment of NF-1-associated spinal deformities is very complex and challenging due to bone dystrophic changes and poor bone stock, making instrumentation very difficult. A meticulous preoperative evaluation and perioperative interdisciplinary approach is mandatory since co-morbidities are very frequent.

## Figures and Tables

**Table 1:** Radiographic features of dystrophic changes in NF-1 patients.

Feature	Location	Definition
Scalloping	Vertebral body	scalloping of the posterior, anterior or lateral wall *
Rotation	Vertebra	rotational deformity as compared to adjacent vertebra
Penciling	Rib	narrowing of the medial portion of the rib **
Wedging	Vertebral body	wedge shaped vertebral body ***
Spindling	Transverse process	transverse process thinned like a spindle
Widening	Spinal canal	enlarged interpedicular distance ****
Enlargement	Neuroforamen	enlarged neuroforamen *****
Soft tissue mass	Paravertebral	seen mostly on MRI

* >3 mm thoracic spine, >4 mm lumbar spine. ** Width of the rib is smaller than that of the narrowest portion of the second rib. *** Similar to congenital hemivertebra. **** Seen in the ap projection compared to the adjacent vertebra. ***** Seen in the lateral view compared to the adjacent vertebra.

**Table 2:** Results according to type of initial surgical procedure.

Type of Index Surgical Procedure	Age at Surgery *	Duration of Follow-Up *	Curve ° Pre-Op	Curve ° Last Follow-Up	% Curve Correction	T1-12 Postoperative †	T1-12 Last Follow-Up †	Growth T1-12 ‡
Growth-preserving	7.7 (±2.3)	6.7 (±3.6)	77 (±11.4)	33.1 (±10.6)	54.1 (±14.7)	19.6 (±3)	22.8 (±2.9)	0.73 (±0.17)
Fusion	13.4 (±2.3)	3.1 (±1.7)	66 (±13.6)	26 (±11.3)	66 (±23.7)			

Legend: *—years; °—Cobb angle; T1-12—thoracic spine height; †—in centimeters; ‡—in centimeters per year.

## Data Availability

All medical data are available on request and will be provided if needed by the corresponding author.
